# MicroED Structure of a Protoglobin Reactive Carbene Intermediate

**DOI:** 10.1021/jacs.2c12004

**Published:** 2023-03-22

**Authors:** Emma Danelius, Nicholas J. Porter, Johan Unge, Frances H. Arnold, Tamir Gonen

**Affiliations:** † Department of Biological Chemistry, University of California, Los Angeles, 615 Charles E. Young Drive South, Los Angeles, California 90095, United States; ‡ Howard Hughes Medical Institute, University of California, Los Angeles, Los Angeles, California 90095, United States; § Division of Chemistry and Chemical Engineering, California Institute of Technology, 1200 East California Boulevard, MC 210-41, Pasadena, California 91125, United States; ∥ Department of Physiology, University of California, Los Angeles, 615 Charles E. Young Drive South, Los Angeles, California 90095, United States

## Abstract

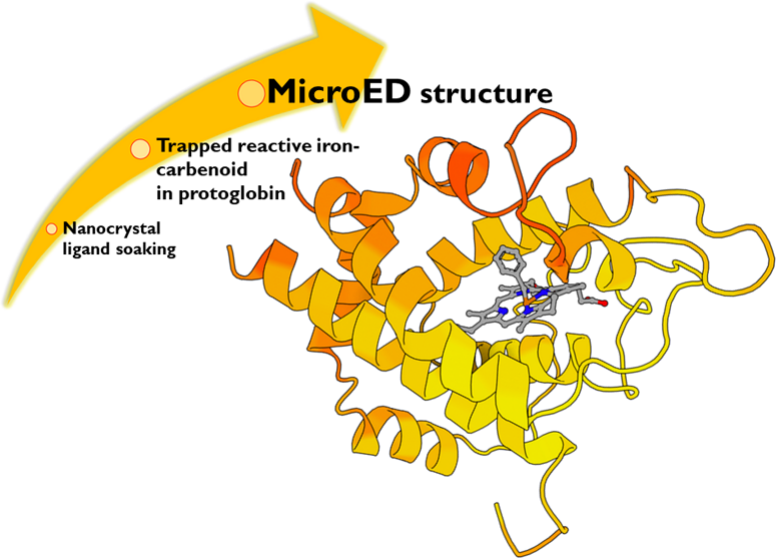

Microcrystal electron diffraction (MicroED) is an emerging technique that has shown great potential for describing new chemical and biological molecular structures. Several important structures of small molecules, natural products, and peptides have been determined using *ab initio* methods. However, only a couple of novel protein structures have thus far been derived by MicroED. Taking advantage of recent technological advances, including higher acceleration voltage and using a low-noise detector in counting mode, we have determined the first structure of an *Aeropyrum pernix* protoglobin (*Ape*Pgb) variant by MicroED using an AlphaFold2 model for phasing. The structure revealed that mutations introduced during directed evolution enhance carbene transfer activity by reorienting an α helix of *Ape*Pgb into a dynamic loop, making the catalytic active site more readily accessible. After exposing the tiny crystals to the substrate, we also trapped the reactive iron-carbenoid intermediate involved in this engineered *Ape*Pgb’s new-to-nature activity, a challenging carbene transfer from a diazirine via a putative metallo-carbene. The bound structure discloses how an enlarged active site pocket stabilizes the carbene bound to the heme iron and, presumably, the transition state for the formation of this key intermediate. This work demonstrates that improved MicroED technology and the advancement in protein structure prediction now enable investigation of structures that was previously beyond reach.

## Introduction

The identification of novel enzymes through protein engineering and directed evolution has made biocatalysis a competitive tool in modern organic synthesis.^1^ Heme enzymes are particularly interesting due to their ability to form and transfer reactive carbene and nitrene intermediates to effect transformations not known in biology and sometimes not even known in chemical catalysis.^2−4^ Although many new-to-nature heme enzymes have been described, with a wide diversity in their synthetic products, the structural rationale behind these advancements is still missing. Describing the short-lived reactive intermediates of these reactions is of great interest for the development of future biocatalysts, but this has proven very challenging. Using X-ray crystallography, only two carbene-bound intermediates have been reported: a carbene-bound *Rhodothermus marinus* cytochrome c variant^5^ and a myoglobin heme-iron-carbenoid complex, which was observed in a nonreactive configuration.^6^ In many cases, large, well-ordered crystals of the enzymes and their complexes are not accessible, so other methods able to handle much smaller crystals are needed.

MicroED is a cryo-electron microscopy (cryo-EM) method, which has been developed during the last decade^7−9^ and has contributed many structures ranging from small molecules^10^ and peptides^11^ to both soluble^12^ and membrane proteins.^13^ In MicroED, continuous rotation electron diffraction data is collected from three-dimensional crystals using a transmission electron microscope (TEM) under cryogenic conditions. The crystals are typically a billionth the size of crystals used for X-ray diffraction; hence, structures of new and important targets which have been out of reach due to challenges in crystal growth can be determined.^14^ As in X-ray diffraction experiments, the intensities of the diffracted beams are directly recorded, while the phases also used to model the crystal content need to be derived by other means. At atomic resolution, phases can be estimated directly from the intensities computationally by *ab initio* methods. Several novel small molecules, peptides, and natural products have been solved by MicroED using *ab initio* phasing, including the sub-ångström structure of the prion proto-PrPSc peptide,^15^ the antibiotic macrocycle thiostrepton^10^^10^ and the chemotherapeutic teniposide.^16^ Further, radiation damage-induced phasing has been shown previously for MicroED data,^17^ and isomorphous replacement, while theoretically possible, has yet to be demonstrated effectively. Recently, even for macromolecular structures, *ab initio* phasing was demonstrated with the sub-ångström resolution structure of triclinic lysozyme.^18^ However, like with X-ray crystallography, the most common method to derive initial phases for macromolecular MicroED structure determination is molecular replacement (MR), which relies on a starting homologous model. The model is typically a similar protein with a known structure, and the phases can be calculated after its position and orientation are found within the crystal. Due to the growing number of known structures deposited to the PDB as well as computational improvements, MR usage has increased from 50% in 2000 to 80% in 2022, and as such, MR is the first choice in most cases for both X-ray crystallography and macromolecular MicroED. A couple of novel protein structures have been solved by MicroED with MR using the known structure of the wild-type homologue, including a novel mutant of the murine voltage-dependent anion channel at 3.1 Å resolution,^19^ and the 3.0 Å structure R2lox,^20^ but MR remains challenging in cases where structures of closely related homologues are not available.

Recent advances in protein structure prediction can enable MR where experimentally determined structures fail or otherwise are unavailable. The possibility to generate *ab initio* models without closely related homologues took a leap in the 14th Critical Assessment of Structure Prediction (CASP14) with the emergence of the deep learning method implemented in AlphaFold2. On the provided test sets, the peptide backbone atom positions could be predicted accurately to within 1 Å. This accuracy meets the requirement for MR when the diffracting resolution is better than 3 Å.^21^ AlphaFold2 or RosettaMR has already been used to generate a starting model for successfully phasing X-ray data, where no experimental structure was available.^22−24^ However, this approach has not been successfully applied to MicroED data.

Here, we present the previously unknown structure of *Aeropyrum pernix* protoglobin (*Ape*Pgb) determined by MicroED in two different states: resting state and with the reactive intermediate carbene bound following chemical activation of the reaction. The *Ape*Pgb structure described herein is an engineered variant for which no wild-type structure has been experimentally determined. The crystals formed as long and thin plates that were brittle and challenging to isolate; despite significant efforts, the structure could not be obtained by synchrotron X-ray crystallography as very weak, or no diffraction was observed. The structure was obtained using the latest MicroED technology, including a cryo-TEM operating at 300 kV acceleration with parallel illumination, data collection on a direct electron detector operating in counting mode, and cryogenic preservation. The resting state structure was solved by molecular replacement against a computationally generated model from AlphaFold2. As compared to our previously described protoglobin (7UTE), we have added additional data and, after further refinement, could resolve loops B and C. Following exposure of the crystals to reaction-like conditions, the same methodology was used to capture and determine the structure of the carbene-bound reactive intermediate of *Ape*Pgb by MicroED. This is, to the best of our knowledge, the first example of a protein structure bound to an aryl-carbene intermediate. This demonstrates the feasibility of using *ab initio* generated protein models for MR in MicroED at the resolution most well represented by protein structures in the PDB and that MicroED can now contribute novel protein structures, including those of short-lived reactive intermediates that were previously beyond technological reach.

## Results and Discussion

**Figure 1:**
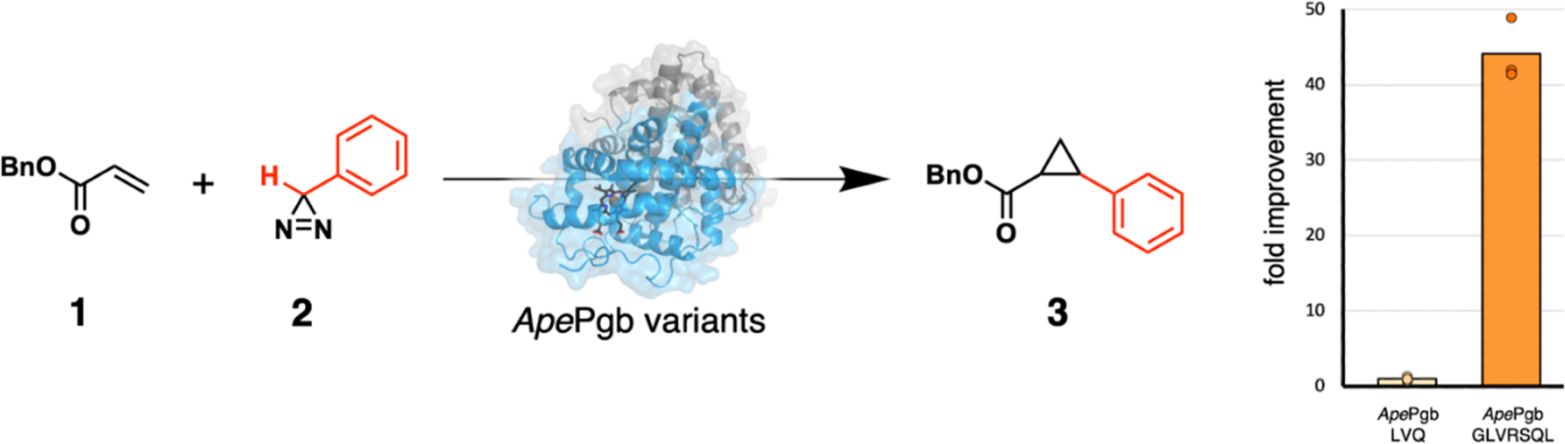
Directed evolution of *A. pernix* protoglobin (*Ape*Pgb) converted this gas-binding protein into an enzyme catalyzing cyclopropanation of benzyl acrylate **1** using phenyldiazirine **2** as a carbene source to generate cyclopropane **3**. The installation of the 4 mutations shown here, introduced during directed evolution, resulted in a >40-fold increase in activity (right; *Ape*Pgb LVQ = *Ape*Pgb W59L Y60V F145Q and *Ape*Pgb GLVRSQL = *Ape*Pgb C45G W59L Y60V V63R C102S F145Q I149L). The reaction scope was further extended to include N–H and Si–H insertion reactions.^27^

Protoglobins are small dimeric heme proteins found in Archaea that are presumed to naturally function as gas binders/sensors.^25^ These proteins have recently gained attention as engineered carbene transfer biocatalysts that can use either diazo compounds^26^ or diazirines^27^ as carbene precursors. Notably, the recent report of diazirine activation (Figure 1) represents the first example of catalytic activation and subsequent carbene transfer from these species. Characterizing the structural details underlying these laboratory-evolved functions can provide deeper insights to guide the future engineering of such biocatalysts.

**Figure 2:**
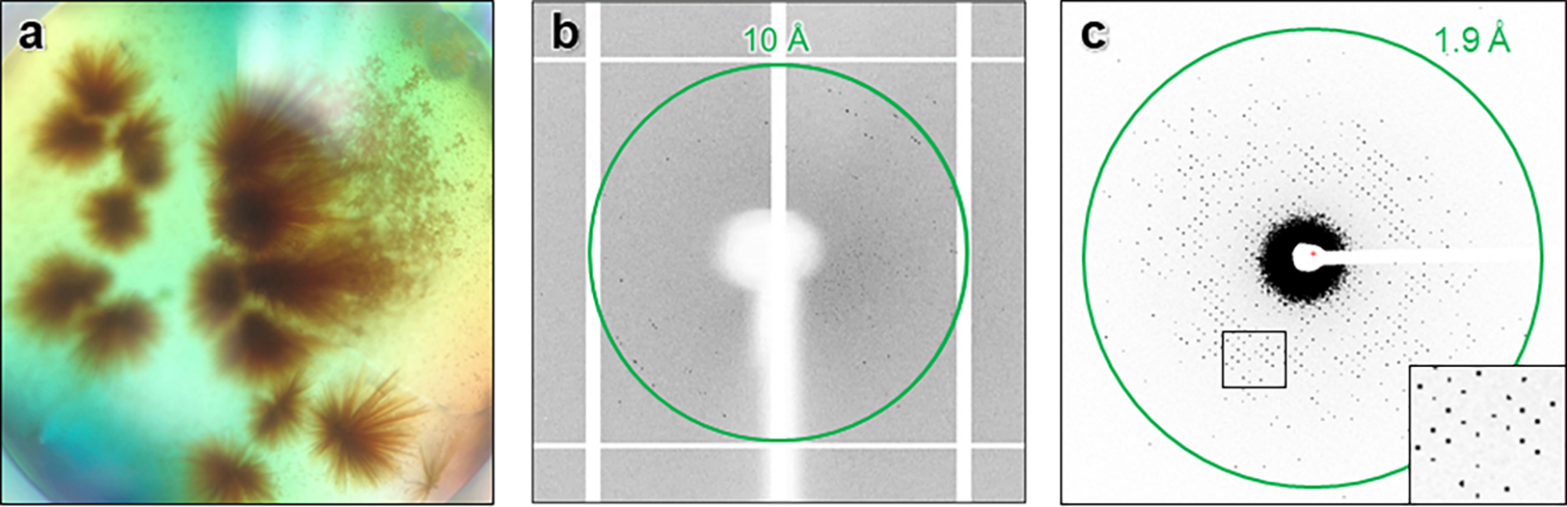
(a) Crystal drop of *Ape*Pgb GLVRSQL in 0.4 M sodium phosphate monobasic/1.6 M potassium phosphate dibasic, 0.1 imidazole (pH 8.0), 0.2 M NaCl. (b) XRD, single exposure. (c) MicroED, single exposure. The green circle indicates levels of resolution.

The *Ape*Pgb variant GLVRSQL described here was expressed and purified as reported previously.^27^ Over 500 conditions were screened for crystal formation identifying only one condition that yielded crystals. To interrogate the structural basis for the gain of cyclopropanation activity (Figure 1), we attempted to determine the crystal structure by X-ray diffraction (XRD). However, the crystals were extremely thin and brittle plates that formed in large clusters (Figure 2a), making it difficult to isolate a single and intact crystal for XRD. While screening, isolated crystals diffracted weakly to around 10–12 Å resolution (Figure 2b), proving insufficient for any structural determination. Crystal optimization assays failed to yield better crystals for XRD despite significant efforts. Instead, the plate-like crystals were prepared for MicroED as described below.

### MicroED Grid Preparation and Diffraction Screening

To examine the crystals in the cryo-TEM, the crystalline clusters were broken into smaller crystallite fragments by perturbation using a pipette, and the remaining crystal slurry was transferred to TEM grids inside of a vitrification robot at 4 °C and 90% humidity. The grids were blotted from the back, vitrified by plunging into liquid ethane, and loaded into a Thermo Fisher Talos Arctica under cryogenic conditions for screening. The crystals appeared as thin sheets on the grids under low magnification (Figure S1). Initial diffraction data were collected on a Ceta-D detector as a movie and processed according to standard MicroED procedures.^28^ However, these plate-like crystals adopted a preferred orientation on the grid, and they had the low symmetry P1 space group that resulted in low completeness, and the overall data quality was insufficient for structural determination.

### MicroED Data Collection

The data quality was dramatically improved by turning to higher acceleration voltage (300 kV) and parallel illumination at the Thermo Fisher Titan Krios TEM, and by collecting the data on the Falcon-4 direct electron detector operating in counting mode, which provides a significantly lower background and higher signal-to-noise ratio.^18^ Whereas scintillator-based cameras, such as the Ceta-D used initially, record the data using integrating mode where the number of electrons is determined by the charge accumulated in a pixel during a readout cycle, direct electron detectors such as the Falcon-4 can be used in counting mode where they detect individual electrons, leading to increased accuracy and higher data quality. The higher acceleration voltage also allowed us to interrogate slightly thicker crystals, further enhancing the signal. Compared with the Ceta-D, the increased sensitivity of the Falcon-4 detector allows more information to be recorded for an identical exposure. In addition, with a faster readout, more fine-sliced data can be collected, further reducing the background for high-resolution reflections. In this case, 840 frames were collected from each crystal on the Falcon-4, as compared with only 160 frames on the Ceta-D for the same exposure. The crystals were continuously rotated (0.15° s^–1^) in the electron beam during the exposure, covering the complete angular range of the stage. The merged data from these experiments yielded about 75% completeness with reasonable merging statistics, even with P1 symmetry (Table S1). The continuous-rotation MicroED data were converted to SMV format using an in-house software that is freely available.^29^

### MicroED Data Processing

Data were indexed and integrated in XDS, as described previously for MicroED.^18^ The integrated data were indexed in space group P1 with unit cell dimensions (*a*, *b*, *c*) = 46.2, 58.3, 80.7 Å, and angles (α, β, γ) = 104.1, 98.6, 90.1°. Scaling and merging in AIMLESS^30^ yielded a data set to 2.1 Å resolution with an overall completeness of ∼75% at a CC_1/2_/*R*_merge_ of 96.5/0.19. Initially, phasing was attempted by MR using the structure of a homologue of *Ape*Pgb GLVRSQL, Y61A *Methanosarcina acetivorans* protoglobin (*Ma*Pgb Y61A; 56% identity, PDB 3ZJI).^31^ However, when no reasonable solution was obtained with this starting model, we redirected our efforts toward predicted models; the sequence of *Ape*Pgb GLVRSQL was subjected to structure prediction with AlphaFold2 using the ColabFold environment.^32^ The generated model was then used as a search model in Phaser to provide a preliminary phase solution for the MicroED data. The best solution with an LLG value of >1300 found 4 monomers in the unit cell. Atomic models were refined with electron scattering factors in Phenix Refine^33^ using automated solvent modeling. Several rounds of refinement resulted in a *R*_work_/*R*_free_ of 0.19/0.22. In addition to the protein with the expected heme groups, the final model includes an imidazole molecule bound to the Fe of the heme in each chain, as well as about 170 water molecules.

### Structure Analysis *Ape*Pgb GLVRSQL

**Figure 3:**
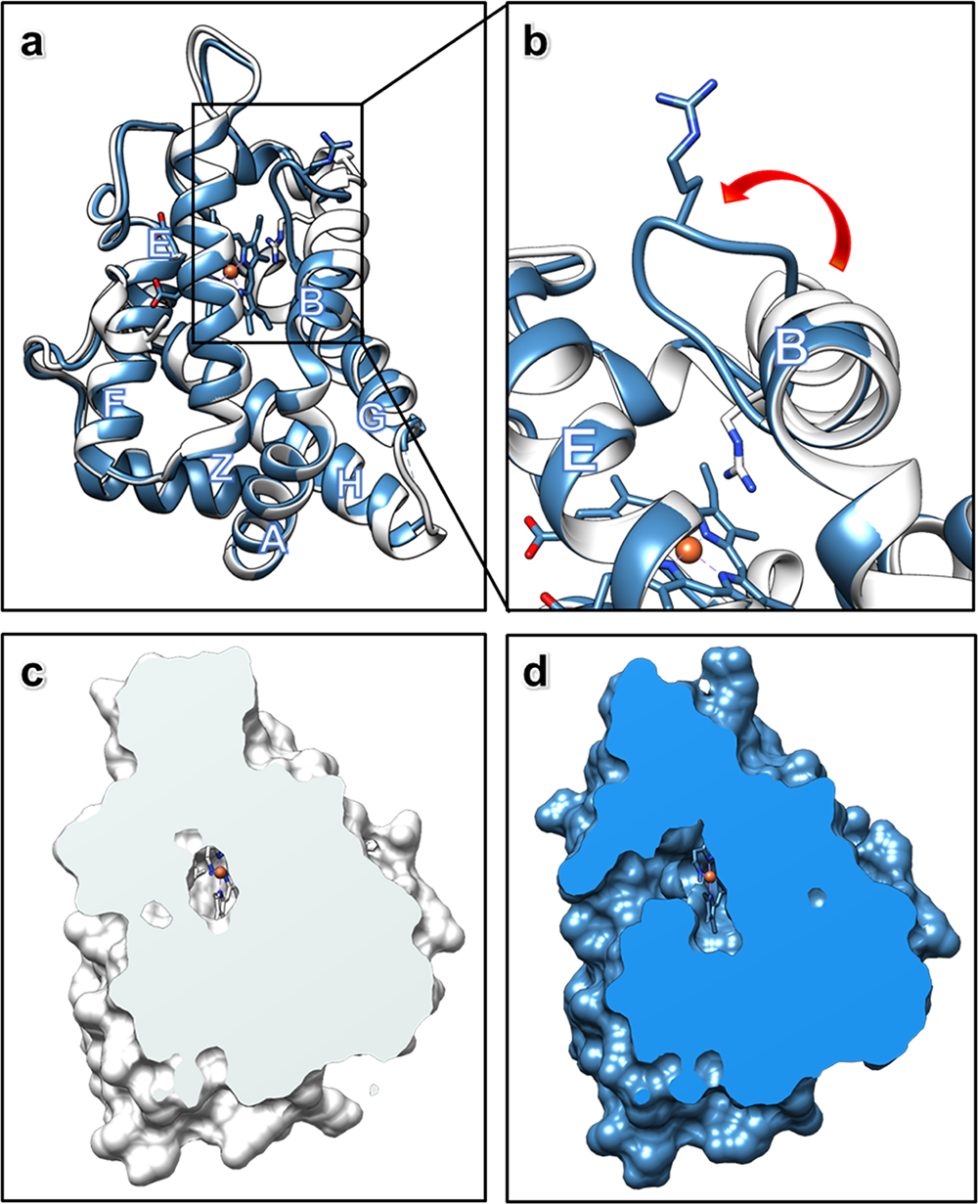
MicroED structure of *Ape*Pgb GLVRSQL: (a) Superposition of the structures of *Ape*Pgb GLVRSQL (blue) with the AlphaFold2 model (white), with helices labeled according to convention for the protoglobin fold. (b) Close-up of the structures of *Ape*Pgb GLVRSQL (blue) with the AlphaFold2 model (white) showing the unwinding of the B helix into a dynamic loop, creating a larger cavity around the active site with increased access to the heme. (c, d) Clipped surface of the AlphaFold2 model (white) and *Ape*Pgb GLVRSQL (blue) showing the effects of the unwinding of the helix and the rearrangement of the B/G interface leading to easier substrate access from outside in addition to the increased space available at the heme.

The structure of *Ape*Pgb GLVRSQL (Figure 3) has 7 mutations installed during directed evolution as compared to the wild-type sequence: C45G, W59L, Y60V, V63R, C102S, F145Q, and I149L. Most of the mutations are near the active site and affect the internal surface. The structure adopts an expanded version of the 3/3 helical sandwich typical of “classical” globins, with an additional N-terminal extension followed by the Z-helix, helping the formation of the homodimer.^31^ The dimer is built by the G- and H-helices, creating a four-helix bundle for the two subunits. The alignment of the AlphaFold2 model and *Ape*Pgb GLVRSQL is presented in Figure 3a, and the alignment of the closest homologue *Ma*Pgb Y61A with the sequence alignment is presented in Figure S2. The *ab initio* model from AlphaFold2 resulted in slightly smaller overall differences to *Ape*Pgb (r.m.s.d. 1.57 Å versus 1.90 Å for *Ma*Pgb Y61A, for details, see Table S3 and Figure 2). When compared to the standard protoglobin fold observed in *Ma*Pgb Y61A, the major difference is the disruption of the B helix between residues 60 and 70. These residues adopt a rigid helical conformation in AlphaFold2 and *Ma*Pgb but are found to be restructured as a loop in *Ape*Pgb GLVRSQL (Figure 3b). Given that the Y61A mutation in *Ma*Pgb does not alter the helical conformation of this region and there is no substantial deviation in the wild-type sequences before the B helix terminus, it is reasonable that this helix would still be present in wild-type *Ape*Pgb. For the *Ape*Pgb GLVRSQL structure with four chains (A–D), the backbone of the disrupted B helix 60–70 is observed for chains A–C, but the density is substantially weaker than surrounding regions (Figure S3). In chain D, residues of the disrupted B helix 60–70 could not be modeled. The weaker electrostatic potential map, the slight differences between chains (Figure S3), and the *B*-factors of the loop (Figure S4) suggest that this region is flexible and thus might be able to adopt different conformations in solution. It is likely that the observed structural change stems from the mutation V63R, which resulted in a 14-fold boost in product yield for the cyclopropanation reaction (Figure 1), the largest improvement from any single mutation during enzyme engineering.^27^ In *Ma*Pgb, V63 is pointing toward the active site, where the natural substrate is two atoms only. The inclusion of the much bulkier arginine residue in this position is difficult to model within the structure of *Ape*Pgb GLVRSQL without major rearrangements. The AlphaFold2-predicted model of *Ape*Pgb GLVRSQL incorrectly orients R63 into the enzyme active site (Figure 3b), similar to the *Ma*Pgb structure. The limited space at the active site, together with the repulsion effect between the positively charged iron and arginine, could be the reason for breaking up the helical conformation in this region to produce a conformation, where R63 is instead positioned at the surface pointing outward (Figure 3b). Thus, the effects of unfavorable steric and electrostatic interactions between the heme and positively charged arginine side chain are presumed to drive the rearrangement of residues 60–70, truncating the B helix in this variant and expanding the active site cavity (Figure 3c,d).

The solvent-inaccessible heme is buried in the protein matrix (Figure 3c,d). This feature is in contrast to most members of the globin family structures, where about 30% of the heme would be surface-accessible.^25^ In natural protoglobins, the diatomic substrates access the active site through two small orthogonal apolar tunnels defined at the interfaces of the B/E and B/G helices. In *Ape*Pgb GLVRSQL, however, the rearrangement of the final turns of helix B obstructs the B/E tunnel through interactions with the main chain and the W62 side chain, resulting in a broadening of the B/G tunnel (Figure 3d). This larger tunnel is presumed to increase diffusion in the active site and allow the entry of larger ligands than the natural diatomic substrates, such as the diazirine and acrylate substrates, targeted in directed evolution (Figure 1). In fact, the bulky benzene moiety is too large to fit into the tunnels present in the AlphaFold2 model, and it seems likely that the drastic expansion of the access pathway is necessary for the passage of the substrate. Molecular dynamics simulations suggest that F145 controls the accessibility of B/G tunnel, though this has yet to be validated experimentally.^25^ The corresponding mutated amino acid Q145, as well as L149, in the engineered *Ape*Pgb lines the expanded tunnel and could reasonably affect the affinity or orientation of the substrate. The F145Q mutation remained throughout the directed evolution of the enzyme despite screening of mutations at this position, and I149L doubled the biosynthetic yield of cyclopropane **3**, underscoring the importance of these mutations to the new activity. The mutations G45 and S102 are both located at the surface of the protein. These mutations remove the cysteine residues in the wild-type sequence. Since these cysteines are located close to one another in space in *Ma*Pgb (5.6 Å Cα–Cα separation), they are potentially capable of forming a disulfide between the A and E helices in wild-type *Ape*Pgb.

### Data Collection of Substrate-Bound *Ape*Pgb GLVRSQL

Nanocrystals allow efficient and homogeneous diffusion of small molecules, giving a fast and convenient way for the determination of ligand-bound complexes, in contrast to a time-consuming and often inaccessible co-crystallization approach. This is especially essential for ligands or intermediates with a limited half-life in solution. Hence, MicroED shows potential for structural determination of reactive intermediates in enzyme-catalyzed reactions. To investigate the reactive intermediate of the reaction shown in Figure 1, the crystal fragments were soaked with carbene precursor **2** (phenyldiazirine, Figure 1) according to previously described protocols.^5^ Following 15 min of soaking and the addition of sodium dithionite to mirror reaction-like conditions, the grids were prepared, and data were collected, as described above. The integrated data in this case were indexed in space group P121 with unit cell dimensions (*a*, *b*, *c*) = 58.15, 45.89, 71.71 Å, and angles (α, β, γ) = 90.00, 105.42, 90.00°. Scaling and merging in AIMLESS^30^ to 2.5 Å resolution gave a data set with overall completeness of 72% and a CC_1/2_/*R*_merge_ of 0.97/0.23. The data were phased by molecular replacement in Phaser using chain A of the *Ape*Pgb GLVRSQL described here. The solution found was top-ranked with an LLG of 4002, containing 2 monomers in the asymmetric unit. The structure was further refined in Phenix Refine^33^ to a *R*_work_/*R*_free_ of 0.23/0.28. The final model was derived by altering the angle and distance describing the carbene interaction until the lowest *R*_free_ value was obtained.

### Structure Analysis of the Metallo-Carbene Structure

**Figure 4:**
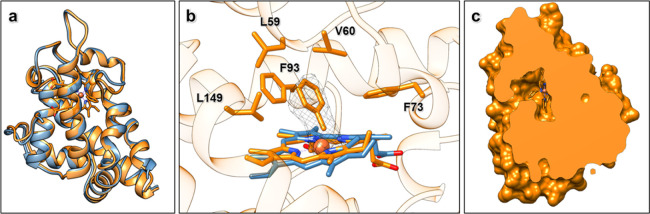
MicroED structure of carbene-bound *Ape*Pgb GLVRSQL. (a) Superposition of the structures of *Ape*Pgb GLVRSQL (blue) and the carbene-bound intermediate (orange). (b) Polder omit map (2.5σ) for the metallo-carbene complex (orange), also including the heme from unbound *Ape*Pgb GLVRSQL (blue) for comparison. (c) Clipped surface of the carbene-bound *Ape*Pgb GLVRSQL, showing a decrease in the size of the B/G channel as compared to the unbound *Ape*Pgb GLVRSQL.

Carbene transfer from a diazirine is thought to involve the formation of a putative reactive iron heme-carbene intermediate, which transfers the carbene to a second substrate, followed by product release and regeneration of the catalyst.^27^ In the metallo-carbene structure described here, the observed overall fold for the carbene-bound *Ape*Pgb GLVRSQL is the same as for the unbound, with the only small differences observed in the 60–70 loop region (Figure 4a). Interestingly, the MicroED density of this loop is a little more defined than in unbound *Ape*Pgb, which might indicate that the loop rigidifies upon substrate binding (Figure S5). In particular, residue W62 is better described by density. *Ape*Pgb GLVRSQL was engineered for activation of a benzene-substituted diazirine (Figure 1), a much larger molecule than any natural protoglobin substrate. As discussed above, increased diffusion into and out of the active site and accommodation of larger substrates near the heme cofactor likely play a significant role in the improved activity of *Ape*Pgb GLVRSQL. For example, amino acids L59 and V60 are both located in the active site, with their side chains pointing toward the binding area on the distal side of the heme group (Figure 4b). Both the selected mutations W59L and Y60V introduce substantially smaller side chains, forming a larger cavity between the heme and the B helix. The main chain conformations for residues 59 and 60 in *Ape*Pgb GLVRSQL and the AlphaFold2 model are similar, and modeling of the original residues, W59 and Y60, discloses significant steric clashes of such residues with the aryl ring of the carbene, suggesting that there would not be sufficient room for this intermediate in the wild-type protoglobin. Further, the side chain of F93 adjusts slightly to form a pi-stacking interaction with the phenyl group of the carbene (Figure 4b). These intermolecular interactions likely stabilize the binding and orientation of the phenyl carbene, each contributing to the improved reactivity gained through evolution. The observed MicroED density and the occupancy of the carbene suggest a single carbene species as the dominant form, where the rate of carbene formation is greater than the rate of carbene decay in the absence of the second substrate.

While it is clear that mutations in *Ape*Pgb GLVRSQL have reshaped the active site, the heme exhibits a similar ruffled distortion to that observed in *Ma*Pgb and other protoglobins (Figure S2).^25^ Out-of-plane distortions to the porphyrin ring are known to alter the electrostatic and ligand-binding properties of the bound iron,^34^ but the specific changes associated with any specific distortion are challenging to measure and remain unclear. When comparing the *Ape*Pgb GLVRSQL with the carbene-bound intermediate, the heme in the ligand-bound variant is further distorted, as illustrated by measuring the angle deviation from the coordination plane. This comparison of heme ring conformations is given in Figure S7. The position of the carbene that resulted in the lowest *R*_free_ in similar refinement rounds is found at a distance of 1.74 Å (Fe–C1) and at an angle of about 128° (Fe–C1–C2). These values are comparable to the previously determined protein structure describing a heme-carbene complex,^5^ as well as an iron porphyrin X-ray structure in which a diaryl-carbene is bound to the Fe atom.^35^ When comparing the B/G helix interfaces of the unbound and carbene-bound states, it seems that binding the substrate has closed the passage slightly (Figure 4c), which coincides with the observation that the residues around the active site and at the solvent tunnel are less dynamic when the substrate is bound. This change is also observable in the *B*-factor gradient (Figure S4). The efficiency of enzymes in accelerating chemical reactions is explained by both their ability to preorganize the active site for transition state stabilization^36^ as well as sample the conformational ensemble required for substrate binding, reaction, and product release.^37^ For this, some inherent flexibility of the enzyme structure is required. The increase in flexibility observed for *Ape*Pgb GLVRSQL can enable the enzyme to adopt the conformations important for the different processes. The following observed rigidification upon binding the substrate might function to preorganize the active site for transition state stabilization. Notably, donor-substituted carbenes are known to be short-lived and highly reactive.^38^ That such a sensitive intermediate can be trapped and observed by MicroED underscores the value of this technique and the insights it can provide into such systems. The homogeneity of the bound intermediate within the crystal is likely enhanced by the improved diffusion and smaller sample size inherent to microcrystalline samples, providing a better context for the atomic details underlying the enzyme chemistry. The atomic details underlying the engineered carbene transfer chemistry developed in these protoglobins will serve to guide future enzyme engineering, leading to the further development of future biocatalysts.

## Conclusions

In conclusion, comparisons to both the experimental structure of the related *Ma*Pgb as well as the predicted AlphaFold2 model show good overall agreement. It highlights the significance of the disruption introduced into the B helix region of the protoglobin fold and implicates the V63R mutation as a factor in this structural change. The broadening of the active site access tunnel relates well to the increased reaction rates observed for this variant. In modern crystallography, most protein structures are phased by molecular replacement using a related model from the protein structure data bank. To date, structure determination using MicroED in the absence of a reasonable search model has been set back due to the lack of experimental phasing techniques analogous to anomalous scattering in X-ray crystallography. We present the determination of a novel structure that could be solved by molecular replacement made possible by an *ab initio* generated model from AlphaFold2 in concert with higher-quality data accessible due to advanced detector development and a high-voltage electron microscope. We further used this technology to investigate the formation of the reactive metallo-carbene and describe the first structure of an aryl-carbene intermediate in a protein structure. As the crystals used in this study were not amenable to X-ray diffraction, this example adds an important tool for the determination of highly sought protein structures.

## Supporting Information Available

Methods, MicroED crystallographic tables, and supplementary figures (PDF)
