# Supplementary material for: MicroED Structure of a Protoglobin Reactive Carbene Intermediate
Source: J Am Chem Soc. 2023 Mar 22;145(13):7159–65. doi: 10.1021/jacs.2c12004 (PMC10080679; doi:10.1021/jacs.2c12004)
Supplement: Supplementary file 1 — ja2c12004_si_001.pdf [file ja2c12004_si_001.pdf]

MicroED structure of a protoglobin reactive carbene intermediate

Emma Danelius[1,2], Nicholas J. Porter[3], Johan Unge[1], Frances H. Arnold[3], & Tamir Gonen[1,2,4*]

1. Department of Biological Chemistry, University of California Los Angeles, 615 Charles E.Young Drive South, Los Angeles, CA 90095, USA.

2. Howard Hughes Medical Institute, University of California Los Angeles, Los Angeles, CA 90095, USA.

3. Division of Chemistry and Chemical Engineering, California Institute of Technology, 1200 East California Boulevard, MC 210-41, Pasadena, California 91125, USA.

4. Department of Physiology, University of California Los Angeles, 615 Charles E. Young Drive South, Los Angeles, CA 90095, USA.

Supporting Information

Methods

Protein crystallization. The crystal drops used for this study were setup using 0.5 µL of 20 mg/mL protein (50 mM potassium phosphate (pH 8.0), 150 mM NaCl) and 2.5 µL precipitant (0.4 M sodium phosphate monobasic / 1.6 M potassium phosphate dibasic, 0.1 M imidazole (pH 8.0), 0.2 M NaCl). Large crystal clusters appeared after 1-3 days.

Grid preparation, ApePgb GLVRSQL. Quantifoil R 2/2 - 200 copper mesh grids were glow discharged (30 s, 15 mA, negative polarity) and transferred to a Leica GP2 vitrification robot with the sample chamber set to 90% relative humidity and 4 °C. The crystal drop was diluted with 15 µL of mother liquor and the needle-like crystals were broken into smaller pieces by gently pipetting into the drop. The resulting protein crystal slurry (2 µL) was pipetted onto the carbon side of the grid in the vitrification chamber and allowed to incubate for 20 seconds. The grid was blotted from the back for 30 seconds, plunged into liquid ethane and transferred to liquid nitrogen for storage.

Grid preparation, carbene-bound ApePgb GLVRSQL. The crystal drop was diluted with 10 µL 3-phenyl-3H-diazirine dissolved in the crystallization condition, making a final concentration of 30 µM 3-phenyl-3H-diazirine. The needle-like crystals were broken into smaller pieces by gently pipetting into the drop, and the resulting slurry was left at room temperature for 15 minutes. Quantifoil R 2/2 - 200 copper mesh grids were glow discharged (30 s, 15 mA, negative polarity) and transferred to a Leica GP2 vitrification robot with the sample chamber set to 90% relative humidity and 4 °C. Following addition of 50 mM sodium dithionite to the drop, 2 µL of the protein crystal slurry soaked with 3-phenyl-3H-diazirine was pipetted onto the carbon side of the grid in the vitrification chamber and allowed to incubate for 20 seconds. The grid was blotted from the back for 30 seconds, plunged into liquid ethane and transferred to liquid nitrogen for storage.

Data collection. The grids were loaded into a Thermo Fisher Scientific Titan Krios G3i transmission electron microscope (300 kV) under cryogenic conditions. Following screening for crystals at low-magnification in imaging mode, using the Thermo Scientific EPU-D software, identified crystals which appeared as thin rectangular sheets on the grid were tested for initial diffraction using 1 second single exposure in diffraction mode. Well diffracting crystals were setup for data collection; the crystals were brought to eucentric height, and a single 1 second exposure was repeated at the starting tilt angle. The MicroED data were collected using a Falcon 4 direct electron detector in counting mode as a movie with

continuous rotation of the stage at a rate of 0.15° s-1, with the selected area aperture of 100 µm and the beam stop inserted. Frames were read out every 0.5 s giving MRC datasets of 840 images, corresponding to a 60° wedge from each crystal. The total wedge that was collected over several datasets corresponded to approximately +70° to -70°.

Data processing and refinement. The MRC files were converted to individual frames in SMV format using the freely available MicroED software (https://cryoem.ucla.edu/). The reflections were indexed and integrated in XDS. The generated datasets were scaled in AIMLESS and phased by molecular replacement in Phaser. For *Ape*Pgb GLVRSQL a structure of predicted by AlphaFold2 through the ColabFold environment was used as a search model, and for the carbene-bound structure Chain A of *Ape*Pgb GLVRSQL was used as search model. All models were refined in phenix.refine using electron-scattering factors. The statistics are given in Supplementary Data Tables 1 and 2. Molecular graphics and analyses was performed with UCSF ChimeraX, Schrödinger Maestro, and Protein Imager.

*Data availability***.** The coordinates and structure factors for the structures have been deposited to the PDB under accession codes 8EUM and 8EUN. The corresponding density maps has been deposited to the EMDB under codes EMD-28615 and EMD-28616.

Supplementary Data Table 1. MicroED Data collection and refinement statistics, *Ape*Pgb GLVRSQL.

Integration	
Wavelength (Å)	0.0197
Acceleration voltage	300 kV
Temperature	-196°C
Space group	P1
Unit cell (a, b, c) (Å)	46.2, 58.3, 80.7
Unit cell (α = β = γ) (°)	104.1, 98.6, 90.1
R_{merge}	0.19
I/σ(I)	6.13 (2.33)
Completeness (%)	74
$CC_{1/2}$	96.5
Wilson *B*-factor (Å2)	9.14
**Refinement**	
Resolution range (Å)	24.47 - 2.10
Total reflections (no.)	33786
R_{work}	0.188
R_{free}	0.212
No. of atoms	6547
Protein	6160
Ligand/ion	192
Water	195
B-factors	
Protein	28.39
Ligand/ion	21.20
Water	17.29
R.m.s. deviation	
Bonds length (Å)	0.014
Bond angles (°)	1.559

Supplementary Data Table 2. MicroED Data collection and refinement statistics, *Ape*Pgb GLVRSQL metallo-carbene complex.

Integration	
Wavelength (Å)	0.0197
Acceleration voltage	300 kV
Temperature	-196°C
Space group	P121
Unit cell (a, b, c) (Å)	58.15, 45.89, 71.71
Unit cell (α, β, γ) (°)	90.00, 105.42, 90.00
R_{merge}	0.18
I/σ(I)	5.73 (2.6)
Completeness (%)	70
CC1/2	96.2
Wilson B-factor (Å2)	6.8
**Refinement**	
Resolution range (Å)	24.36 - 2.50
Total reflections (no.)	40105
R_{work}	0.235
R_{free}	0.284
No. of atoms	3228
Protein	3104
Ligand/ion	100
Water	64
B-factors	
Protein	20.81
Ligand/ion	16.43
Water	16.34
R.m.s. deviation	
Bonds length (Å)	0.005
Bond angles (°)	0.877

Supplementary Data Table 3. R.m.s. deviation for *Ape*Pgb GLVRSQL Chain A – D, the AlphaFold2 model, and *Ma*Pgb Y61A, obtained using Swiss-PdbViewer for main chain atoms.

	***Ape*Pgb GLVRSQL AlphaFold2 model**	***Ma*Pgb Y61A (PDB ID 3ZJI)**
***Ape*Pgb GLVRSQL Chain A**	1.57	1.90
***Ape*Pgb GLVRSQL Chain B**	1.57	1.90
***Ape*Pgb GLVRSQL Chain C**	1.58	1.90
***Ape*Pgb GLVRSQL Chain D (no loop 62–68)**	0.87	1.30
***Ape*Pgb GLVRSQL AlphaFold2 model**	—	1.06

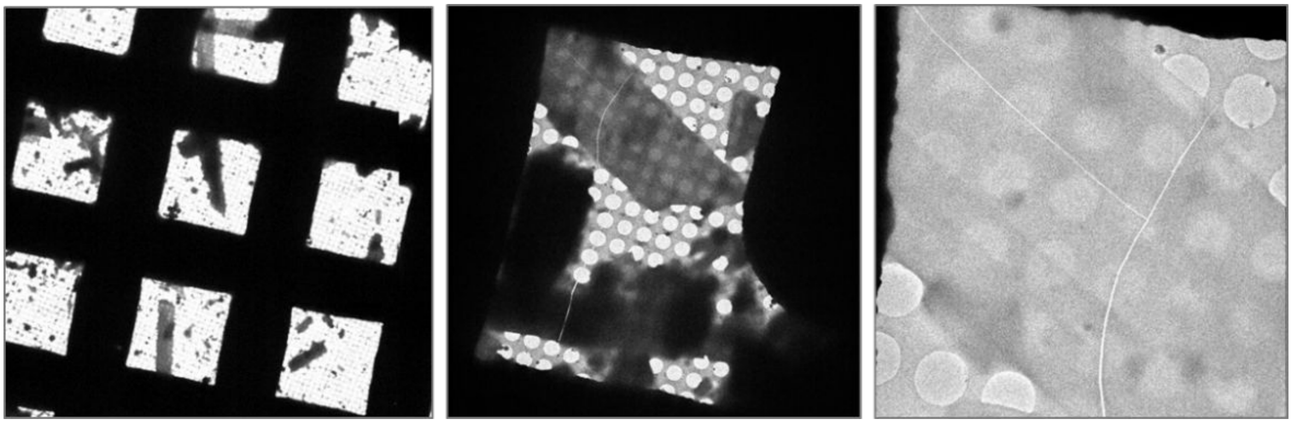

Supplementary Figure 1. The thin plate clusters of *Ape*Pgb GLVRSQL, in the TEM at 210x, 940x, and 3400x magnification.

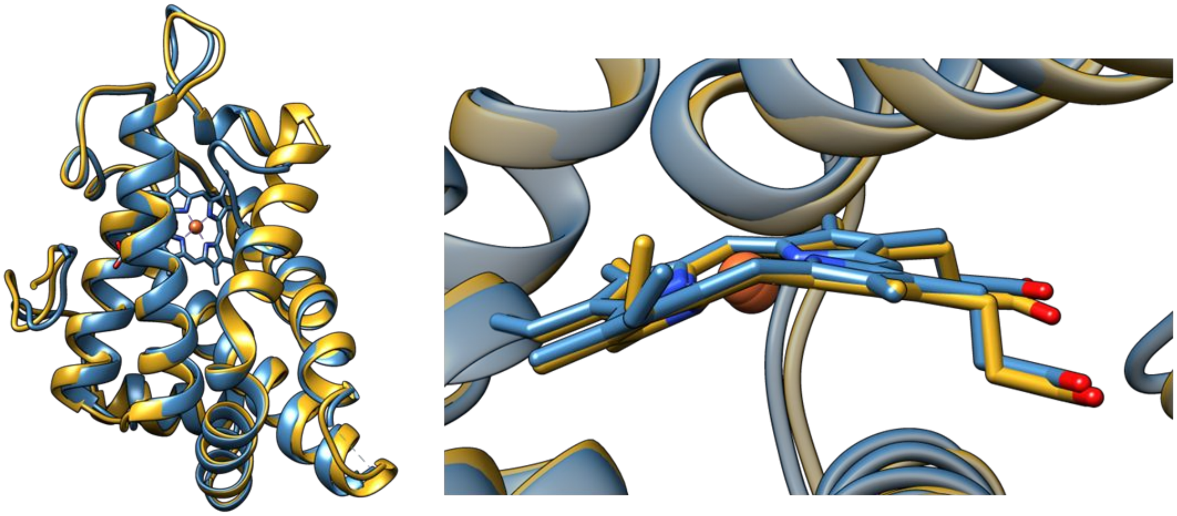

```
CLUSTAL 2.1 multiple sequence alignment


ApePgb    1  MTPSDIPGYDYGRVE-KSPITDLEFDLLKKTVMLGEKDVMYLKKAGDVLK    49
3ZJI      1  MSVEKIPGYTYGETENRAPFNLEDLKLLKEAVMFTAEDEEYIQKAGEVLE    50
             *: ..**** **..* ::*:.  ::.***::**:  :*  *::***:**:

ApePgb   50  DQVDEILDLLVGWRASNEHLIYYFSNPDTGEPIKEYLERVRARFGAWILD    99
3ZJI     51  DQVEEILDTWAGFVGSHPHLLYYFTSPD-GTPNEKYLAAVRKRFSRWILD    99
             ***:****  .*: .*: **:***:.** * * ::**  ** **. ****

ApePgb  100  TTSRDYNREWLDYQYEVGLRHHRSKKGVTDGVRTVPHIPLRYLIAQIYPL    149
3ZJI    100  TCNRSYDQAWLDYQYEIGLRHHRTKKNQTDNVESVPNIGYRYLVAFIYPI    149
             * .*.*:: *******:******:**. **.*.:**:*  ***:* ***:

ApePgb  150  TATIKPFLAKKGGSPEDIEGMYNAWFKSVVLQVAIWSHPYTKENDW        195
3ZJI    150  TATMKPFLARKGHTPEEVEKMYQAWFKATTLQVALWSYPYVKYGDF        195
             ***:*****:*   **::* **:****:..****:**:**.* .*:
```

Supplementary Figure 2. Sequence alignment and superposition of *Ape*Pgb GLVRSQL (blue) and the closest homologue protoglobin (Y61A *Methanosarcina acetivorans* protoglobin *(Ma*Pgb), PDB 3ZJI, yellow*)*. The mutations introduced through directed evolution are highlighted in yellow. The close-up of the heme shows the similar ruffled distortion observed in *Ma*Pgb and other protoglobins.

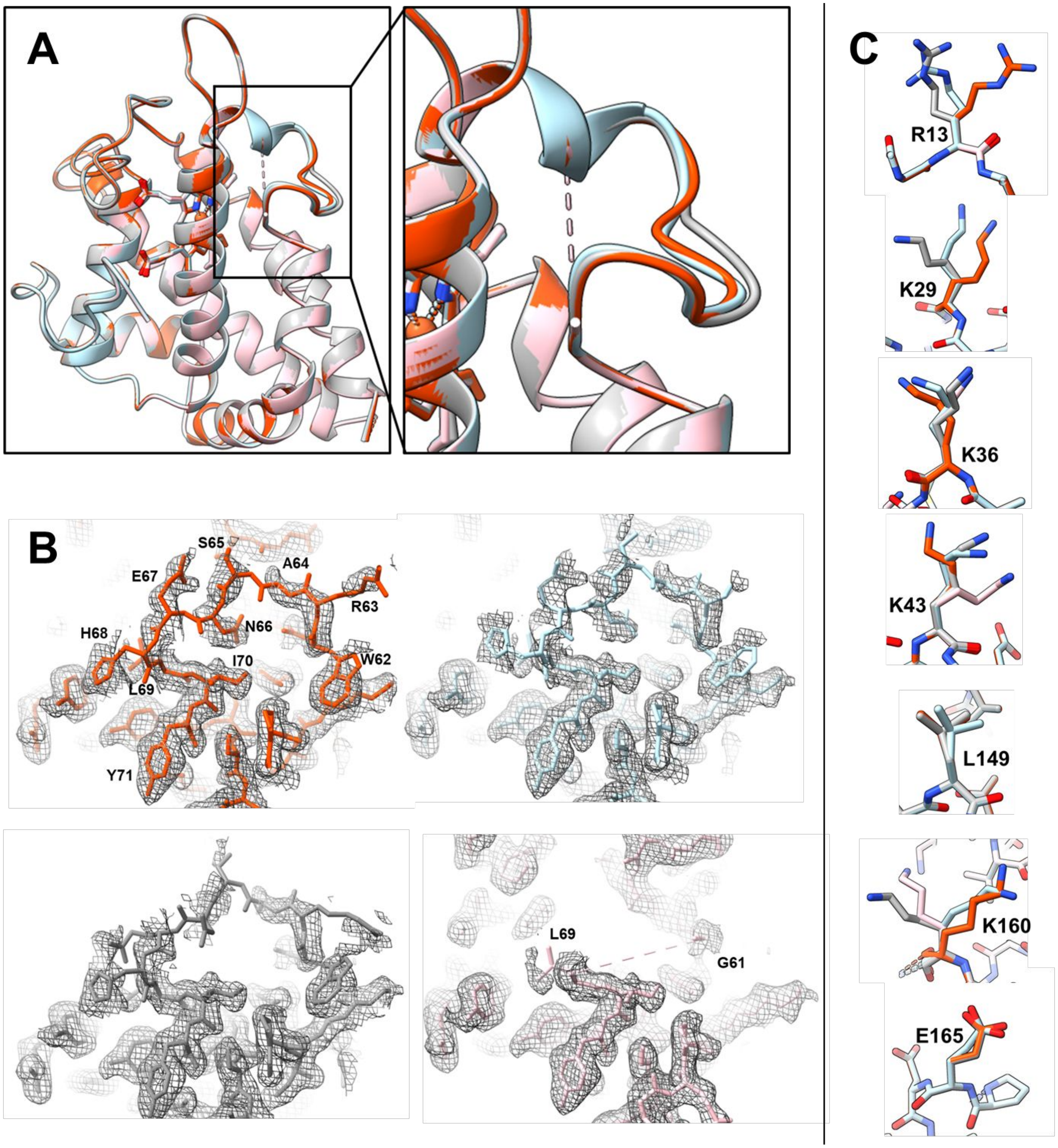

Supplementary Figure 3. Differences of *Ape*Pgb GLVRSQL chain A (red), B (blue), C (grey) and D (pink). A) Overlay of *Ape*Pgb GLVRSQL chain A – D and magnification of the 60–70 loop region, showing the slight differences in backbone fold. B) Density maps and modeled residues for the 60–70 loop region of *Ape*Pgb GLVRSQL. In Chain D there was no density that could be modeled. C) Differences in side chain orientations for *Ape*Pgb GLVRSQL chain A – D for residues not belonging to the 60–70 loop region. No other differences were identified.

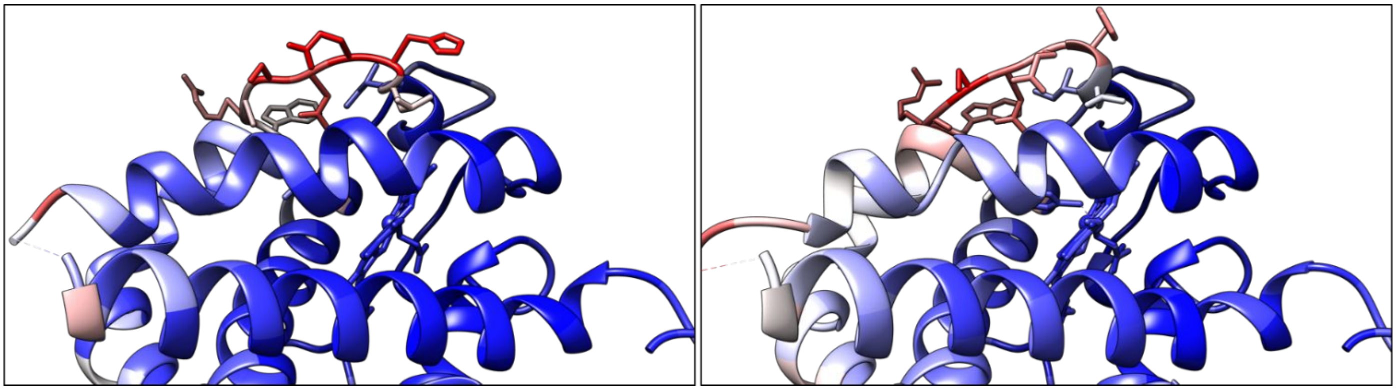

Supplementary Figure 4. *Ape*Pgb GLVRSQL (left) and carbene-bound *Ape*Pgb GLVRSQL (right) showing the average *B*-factors as a color gradient from blue (*B*-factor 20 or less) to red (*B*-factors 50 or more), highlighting the variations within the protein.

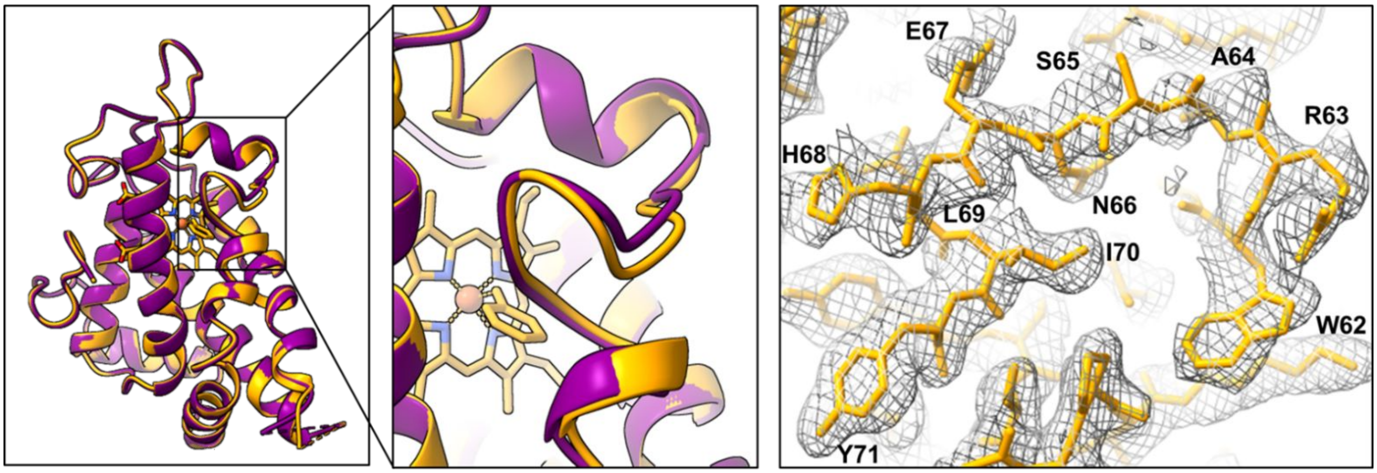

Supplementary Figure 5. Overlay of carbene-bound *Ape*Pgb GLVRSQL chain A (orange) and B (purple), and density map for chain A with modeled residues for the 60–70 loop region.

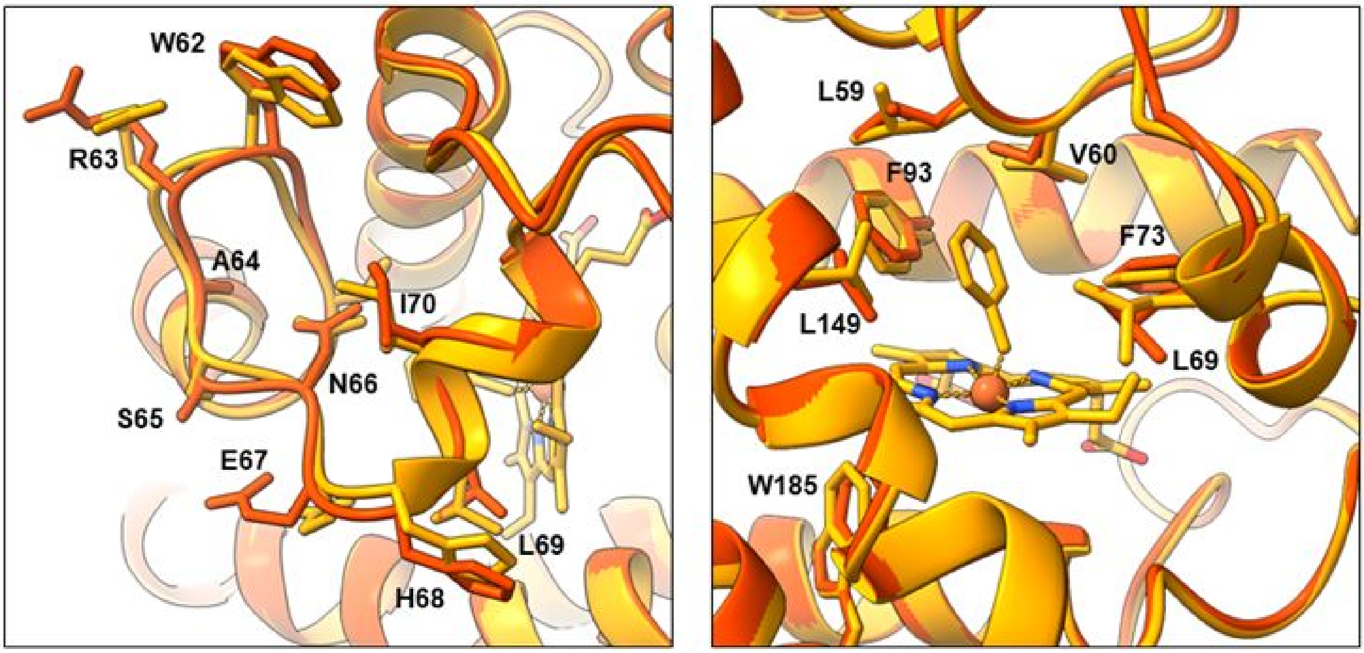

Supplementary Figure 6. Overlay of *Ape*Pgb GLVRSQL chain A (red), and carbene-bound *Ape*Pgb GLVRSQL chain A (orange), showing the 60–70 loop region (left), and the active site (right).

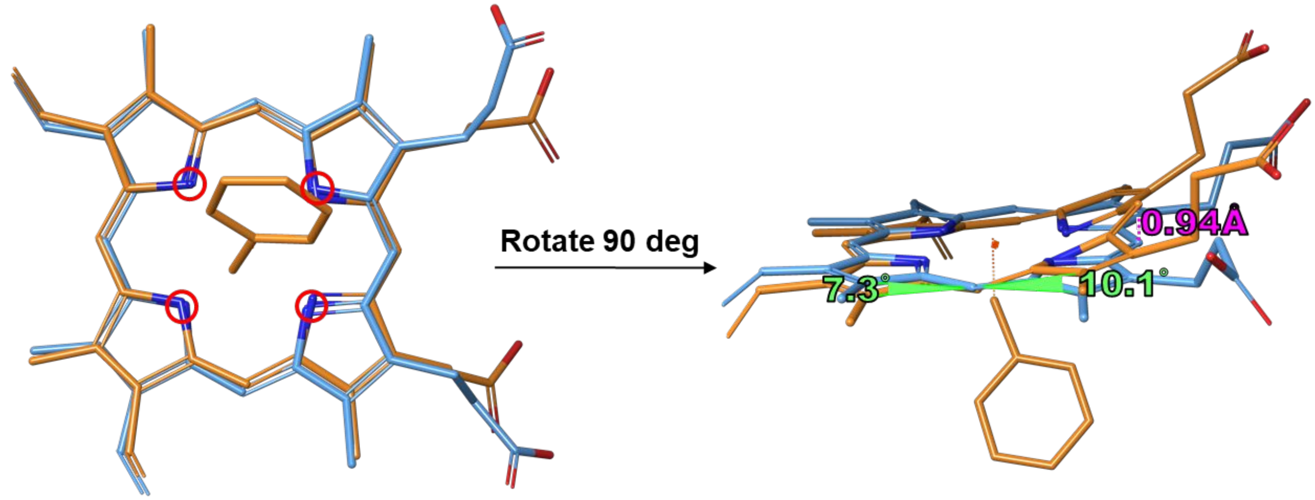

Supplementary Figure 7. Superposition of *Ape*Pgb GLVRSQL (blue) and carbene-bound *Ape*Pgb GLVRSQL (orange), using the metal coordinating atoms as indicated by the red circles, showing up to 10.1˚ difference in heme conformations.
